# A bivalent promoter contributes to stress-induced plasticity of CXCR4 in Ewing sarcoma

**DOI:** 10.18632/oncotarget.11240

**Published:** 2016-08-12

**Authors:** Melanie A. Krook, Allegra G. Hawkins, Rajiv M. Patel, David R. Lucas, Raelene Van Noord, Rashmi Chugh, Elizabeth R. Lawlor

**Affiliations:** ^1^ Translational Oncology Program, University of Michigan, Ann Arbor, MI, USA; ^2^ Department of Pediatrics and Communicable Diseases, University of Michigan, Ann Arbor, MI, USA; ^3^ Department of Pathology, University of Michigan, Ann Arbor, MI, USA; ^4^ Department of Internal Medicine, University of Michigan, Ann Arbor, MI, USA

**Keywords:** CXCR4, epigenetics, plasticity, Ewing sarcoma

## Abstract

Tumor heterogeneity is a major impediment to cancer cures. Tumor cell heterogeneity can arise by irreversible genetic mutation, as well as by non-mutational mechanisms, which can be reversibly modulated by the tumor microenvironment and the epigenome. We recently reported that the chemokine receptor CXCR4 is induced in Ewing sarcoma cells in response to microenvironmental stress. In the current study, we investigated plasticity of CXCR4 expression *in vivo* and assessed whether CXCR4 impacts on tumor growth. Our studies showed that Ewing sarcoma cells convert between CXCR4 negative and CXCR4 positive states *in vivo* and that positive cells are most abundant adjacent to areas of necrosis. In addition, tumor volumes directly correlated with *CXCR4* expression supporting a role for CXCR4 in growth promotion. Mechanistically, our results show that, in ambient conditions where CXCR4 expression is low, the *CXCR4* promoter exists in a poised, bivalent state with simultaneous enrichment of both activating (H3K4me3) and repressive (H3K27me3) post-translational histone modifications. In contrast, when exposed to stress, CXCR4 negative cells lose the H3K27me3 mark. This loss of promoter bivalency is associated with *CXCR4* upregulation. These studies demonstrate that stress-dependent plasticity of CXCR4 is, in part, mediated by epigenetic plasticity and a bivalent promoter.

## INTRODUCTION

Tumor heterogeneity contributes to tumor progression and remains a major challenge in the treatment and diagnosis of cancer as well as for the development of novel cancer therapeutics [1, 2]. Furthermore, although biological heterogeneity between tumor and non-tumor stroma is a major determinant of tumor behavior, it is increasingly evident that phenotypic heterogeneity among tumor cells themselves is also of profound importance to disease progression, therapy response and clinical outcomes. This phenotypic heterogeneity can include variability in gene expression, motility, and metastatic potential across cells in a tumor [3] and can be driven by both genetic and epigenetic mechanisms, as well as by contributions of the tumor microenvironment [2, 4].

The CXCL12-CXCR4 chemotactic axis contributes to metastasis of numerous different human cancers and CXCR4 positive tumor cells are often detected at the leading edge of invasive tumors and in cancer stem cell populations [5, 6]. In light of this, the CXCL12-CXCR4 axis is of great therapeutic interest and pharmacologic approaches are being developed to target CXCR4 signaling as an anti-cancer strategy [7, 8]. We recently showed that expression of CXCR4 is heterogeneous and dynamically regulated in Ewing sarcoma, an aggressive bone and soft tissue tumor that peaks in adolescents and young adults [9]. In particular, exposure of tumor cells to microenvironmental stress, including growth factor deprivation, hypoxia, and physical growth constraints resulted in upregulation of CXCR4, either at the level of mRNA or cell surface protein expression, or both [9]. This upregulation of CXCR4 was associated with phenotypic transition of Ewing sarcoma cells from relatively non-motile states to cells that actively migrated and invaded towards the CXCL12 ligand [9]. Previously, expression of *CXCR4* transcript was found to be increased in tumor biopsies from patients with metastatic Ewing sarcoma compared to localized tumors, suggesting that CXCR4 signaling may contribute to tumor metastasis [10]. In addition, there is evidence that local growth of Ewing sarcomas is also promoted by CXCR4 pathway activation [11]. Thus, elucidation of the mechanisms underlying CXCR4 regulation in Ewing sarcoma could provide insights into the molecular mechanisms of Ewing sarcoma cell heterogeneity and tumor progression.

In the current study we assessed plasticity of CXCR4 in Ewing sarcoma tumor cells *in vivo*. In addition, we evaluated the chromatin state of the *CXCR4* locus in a panel of Ewing sarcoma cell lines to determine if epigenetic plasticity contributes to stress-dependent activation of CXCR4. The findings from these studies demonstrate that Ewing sarcoma cells transition between CXCR4 negative and CXCR4 positive states *in vivo,* that this phenotypic heterogeneity contributes to tumor growth and is, at least in part, driven by epigenetic plasticity of the *CXCR4* promoter in response to microenvironmental stress.

## RESULTS

### Ewing sarcoma cells transition between CXCR4 negative and CXCR4 positive states *in vivo*

**Figure 1:**
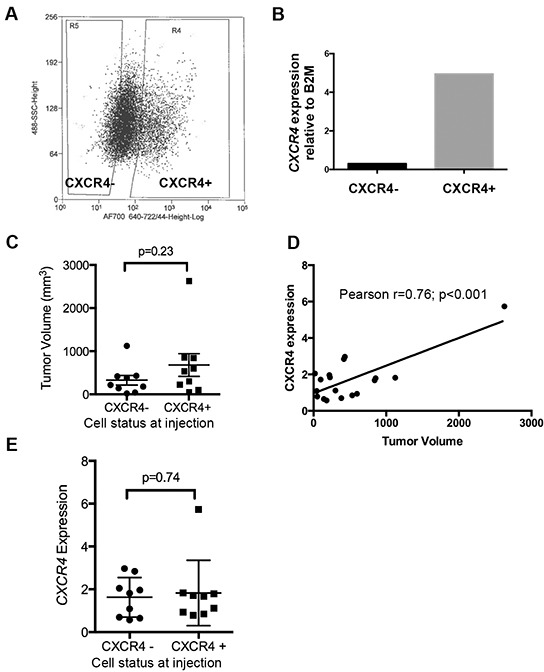
Ewing sarcoma cells transition between CXCR4 negative and positive states *in vivo* **A.** FACS-sorting of TC-32 cells on the basis of cell surface CXCR4 prior to injection into tail veins of NOD-SCID mice. **B.** FACS-sorting generated cell populations with differential expression of *CXCR4* transcript as determined by qRT-PCR. **C.** Tumors were excised at necropsy and volumes determined. Nine tumors were analyzed in each group (from 5 CXCR4 negative and 8 CXCR4 positive recipient mice). **D.** A direct correlation was observed between *CXCR4* expression, as determined by qRT-PCR, and tumor volume in excised tumors. **E.** *CXCR4* expression in tumors at the time of necropsy as determined by qRT-PCR. Expression of *CXCR4* was highly variable. Mean expression was equivalent between the two groups.

**Figure 2:**
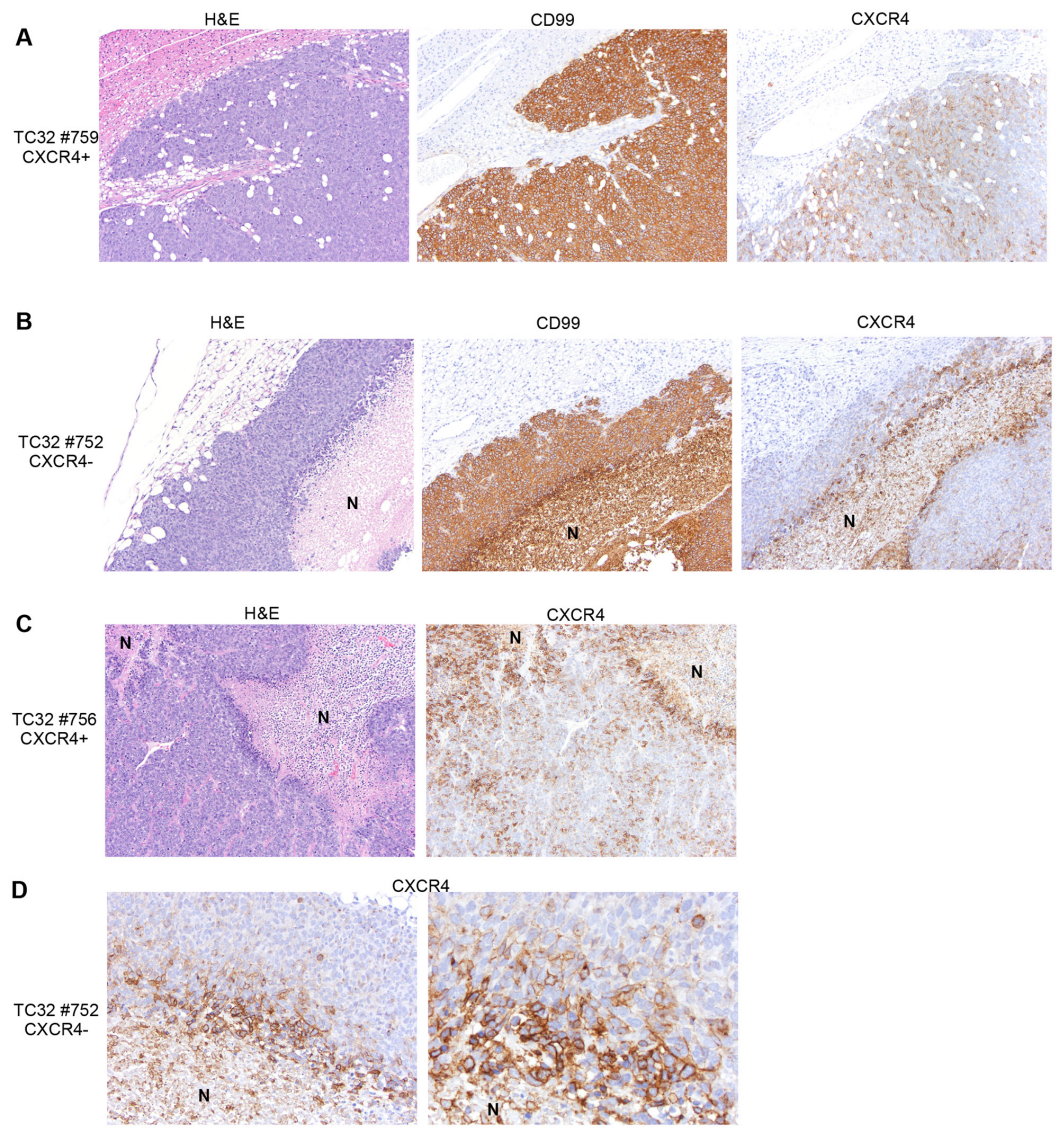
Heterogeneity of CXCR4 expression is evident in tumors irrespective of CXCR4 status at the time of injection **A.** Immunostaining of xenograft tumor sections shows abundant CXCR4 negative tumor cells in tumors derived from CXCR4 positive cells. 10x images of a representative tumor. **B.** CXCR4 positive tumor cells are detected in tumors derived from CXCR4 negative cells. 10x images of a representative tumor. N=necrotic region. **C.** CXCR4 positive cells are increased adjacent to areas of tumor necrosis. 10x images of a representative tumor derived from CXCR4 positive cells (N=necrotic region). **D.** High power images of CXCR4 negative cell-derived tumor as in B (left panel: 20x; right panel: 40x) show increased frequency of CXCR4 positive tumor cells immediately adjacent to areas of necrosis.

We previously showed that Ewing cells transition between CXCR4 positive and CXCR4 negative states *in vitro* and that CXCR4 is induced in response to stress [9]. In order to determine if similar phenotypic transitions occur *in vivo* we FACS-sorted TC-32 cells on the basis of CXCR4 (Figure 1A) and injected cells *via* tail vein into immunodeficient mice. qRT-PCR confirmed that levels of *CXCR4* transcript were substantially lower in the CXCR4 negative cells at the time of cell injection (Figure 1B). Bioluminescence imaging detected no significant difference in time to tumor engraftment between the two groups. After eight weeks, mice were euthanized and tumor numbers and volumes were determined. A total of 5 out of 8 mice developed tumors in the CXCR4- cell population and 8 out of 10 mice developed tumors that were injected with CXCR4 positive cells (p=0.6, Fisher's exact test). Final tumor volumes were also equivalent between the groups at the time of necropsy, with a trend to increased volume in CXCR4 positive cell-derived tumors (Figure 1C). Examination of gene expression in the excised tumors revealed that *CXCR4* transcript levels in resected tumors directly correlated with tumor volume, suggesting that expression of CXCR4 in established tumors might promote tumor growth (Figure 1D). Notably, however, mean *CXCR4* transcript expression in tumors from both groups of recipient mice was equivalent at the time of resection (Figure 1E). Thus, these findings revealed that the CXCR4 state at the time of tumor cell injection was not a key determinant of *CXCR4* expression at the time of tumor resection. Rather, the data suggested that all tumors evolved to a mixed population of CXCR4 positive and CXCR4 negative cells, resulting in a relative decrease in *CXCR4* expression in the CXCR4 positive cohort and an increase in *CXCR4* expression in the CXCR4 negative cohort. To address this possibility, excised tumors were assessed by immunohistochemistry to evaluate the presence of CXCR4 positive and negative tumors cells. Significantly, mixed populations of CXCR4 negative and CXCR4 positive cells were evident in all tumors regardless of their CXCR4 status at the time of injection. In particular, CXCR4 negative/CD99 positive tumor cells were present in abundance in tumors that arose from CXCR4 positive cells (Figure 2A). Conversely, CXCR4 positive/CD99 positive cells were readily detected in tumors that arose from strictly CXCR4 negative cell injections (Figure 2B). Notably, in both groups CXCR4+ cells were most abundant adjacent to areas of necrosis (Figure 2C & 2D). These findings support our prior *in vitro* observations that Ewing sarcoma cells are highly plastic with respect to CXCR4 expression and that exposure to microenvironmental stress promotes acquisition of a CXCR4 positive cell phenotype.

### The *CXCR4* promoter is bivalent in Ewing sarcoma cells

The results of the xenograft studies confirmed that *CXCR4* expression is highly plastic in Ewing sarcoma cells *in vivo* and that transition of cells from CXCR4 negative to CXCR4 positive states is most prominently observed adjacent to areas of necrosis. Given the integral role of epigenetic deregulation in Ewing sarcoma pathogenesis, and our observations that the transition of CXCR4 negative cells into CXCR4 positive states is associated with changes in levels of *CXCR4* transcript, we investigated whether epigenetic plasticity contributes to stress-induced plasticity.

**Figure 3:**
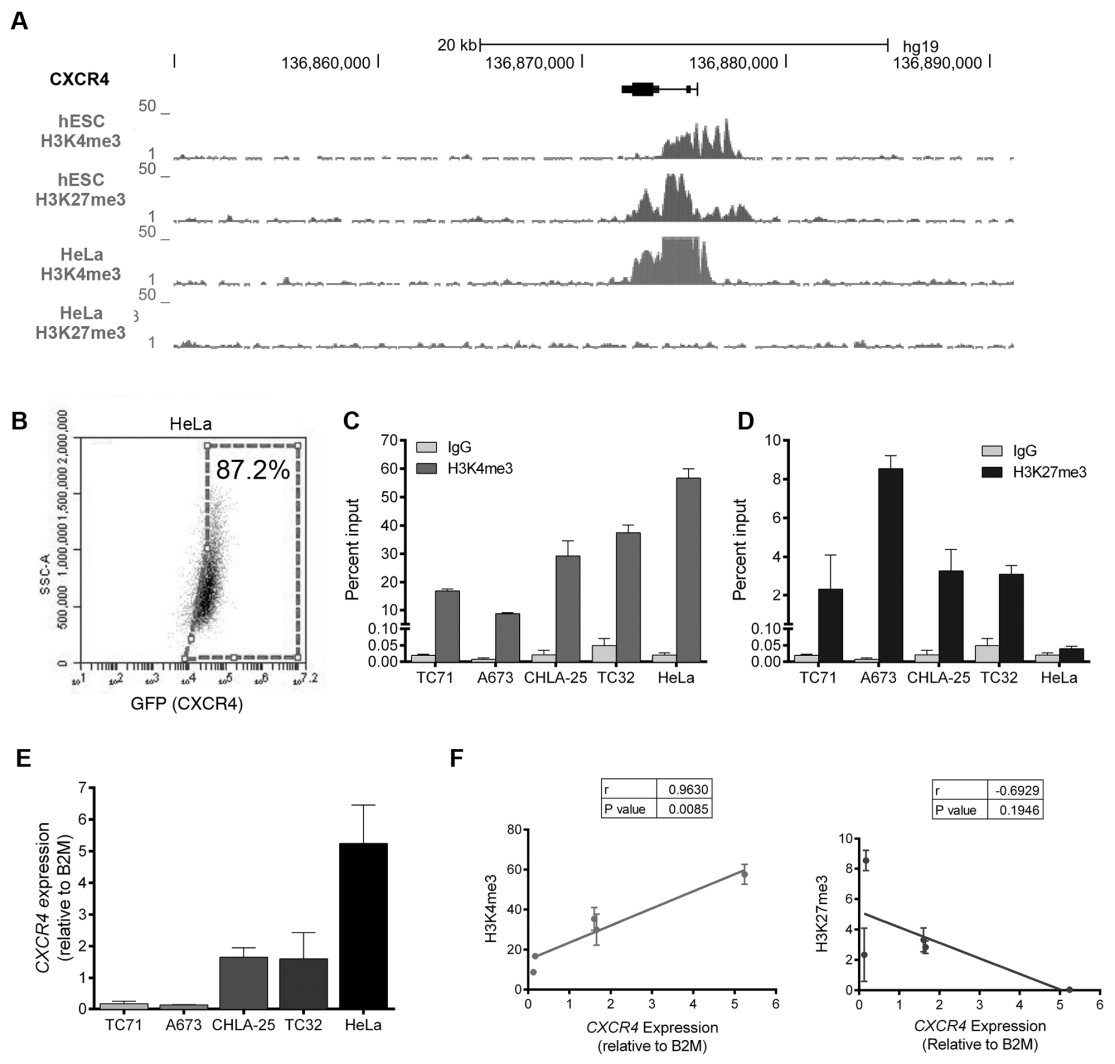
The *CXCR4* promoter is enriched with both the H3K4me3 and H3K27me3 histone marks in Ewing sarcoma cells **A.** Gene tracks for H3K4me3 and H3K27me3 at the *CXCR4* promoter in human embryonic stem cells (hESC) and HeLa cells assembled from the ENCODE database demonstrate a bivalent state in hESC cells and a univalent active state in HeLa cells. **B.** FACS analysis of HeLa cells shows that most cells express CXCR4. **C.** Targeted ChIP-qPCR studies showed that the H3K4me3 is enriched at the *CXCR4* promoter in both Ewing sarcoma and in HeLa cells. **D.** Targeted ChIP-qPCR studies showed that the H3K27me3 modification is enriched at the *CXCR4* promoter of Ewing sarcoma cells but is not present in HeLa cells. **E.** *CXCR4* expression as determined in a panel of Ewing sarcoma cell lines and HeLa cells using qRT-PCR. Data represented as mean ± SEM of three independent experiments. **F.** Expression of *CXCR4* correlates directly with enrichment of H3K4me3 and inversely with H3K27me3 at the *CXCR4* promoter. r= Pearson correlation co-efficient.

Multiple mechanisms contribute to epigenetic regulation of gene expression in normal and malignant development, including post-translational histone modifications at gene promoters [12]. In particular, rapid induction of gene expression in stem cells is achieved by the simultaneous presence of both activating (H3K4me3) and repressive (H3K27me3) histone marks at key developmental gene promoters, creating loci that are silenced but poised for rapid activation in response to appropriate cues [13]. The significance of bivalent loci to gene activation in cancer was recently described in the context of breast cancer stem cells and epithelial-to-mesenchymal transitions [14]. To begin to address whether bivalency might play a role in CXCR4 regulation we first asked whether the *CXCR4* locus is bivalent in embryonic stem cells, cells where bivalency was first described [13]. As shown, analysis of the ENCODE database [15] revealed that both H3K27me3 and H3K4me3 histone modifications are enriched at the *CXCR4* promoter in human embryonic stem cells, consistent with a bivalent state (Figure 3A, top). In contrast, in HeLa cells, the *CXCR4* promoter is characterized by a univalent state, with enrichment of only the H3K4me3 mark and complete absence of the repressive H3K27me3 modification (Figure 3A, bottom). In Ewing sarcoma cell lines we have shown that CXCR4 expression is normally expressed by only a minority of cells under ambient conditions [9]. Conversely, most HeLa cells express high levels of CXCR4 on their cell surface (Figure 3B). To evaluate the chromatin state of the *CXCR4* promoter in Ewing sarcoma we performed ChIP-PCR studies using antibodies directed against the H3K4me3 and H3K27me3 histone modifications and genomic PCR primers specific for the *CXCR4* promoter (Supplementary Figure S1A). These studies revealed that both the activating and repressive histone marks are highly enriched at the *CXCR4* promoter in all Ewing sarcoma cell lines, consistent with the identity of a bivalent locus (Figure 3C & 3D and Supplementary Figure S1B). In contrast, we confirmed that only the active chromatin mark H3K4me3 was detectable at the *CXCR4* promoter in HeLa cells (Figure 3C & 3D and Supplementary Figure S1B). Relative enrichment of the H3K4me3 modification was positively correlated (r = 0.9630) with *CXCR4* expression in the tested cell lines while H3K27me3 was negatively correlated (r = −0.6929) with *CXCR4* transcript expression (Figure 3E).

**Figure 4:**
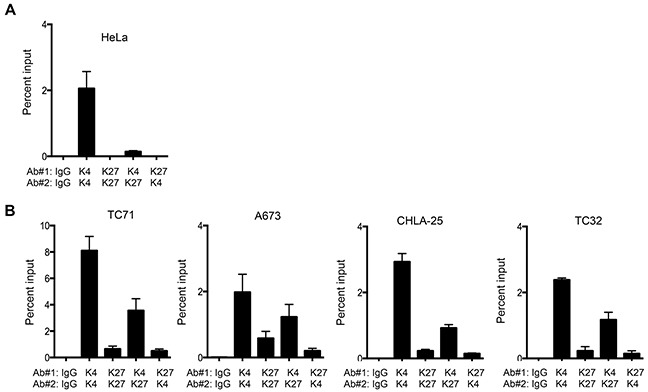
The *CXCR4* promoter is bivalent in Ewing sarcoma cells **A.** Sequential ChIP-qPCR experiments (ChIP-re-ChIP) for H3K4me3 followed by H3K27me3 (K4/K27) and *vice versa* (K27/K4), demonstrated only the H3K4me3 modification in HeLa cells, confirming its univalent chromatin state. **B.** ChIP-re-ChIP of Ewing sarcoma cells showed H3K4me3 at H3K27me3-marked chromatin (K4/K27), and *vice versa* (K27/K4) thus confirming that the *CXCR4* promoter is bivalent. IgG, K4/K4, and K27/K27 served as controls. Data represented as mean ± SEM of three independent experiments.

Our finding that both modifications were enriched at the *CXCR4* promoter in independent ChIP studies was suggestive of, but not conclusive for, bivalency. To more definitively test for bivalency we went on to determine if the simultaneous presence of both marks could be detected in the same chromatin preparation by performing sequential ChIP experiments. As shown, these ChIP-re-ChIP studies again revealed only the presence of the H3K4me3 modification in HeLa cells, confirming its univalent chromatin state (Figure 4A). In Ewing sarcoma samples, H3K4me3 was detected at the *CXCR4* promoter in chromatin that had been isolated by the H3K27me3-directed antibody (K27/K4) and *vice versa* (K4/K27) (Figure 4B). Thus, in ambient, unstressed conditions, the *CXCR4* promoter of Ewing sarcoma cells resides in a bivalent state and, as such, may be poised and ready for activation in response to microenvironmental cues.

### Upregulation of CXCR4 expression is associated with loss of H3K27me3

**Figure 5:**
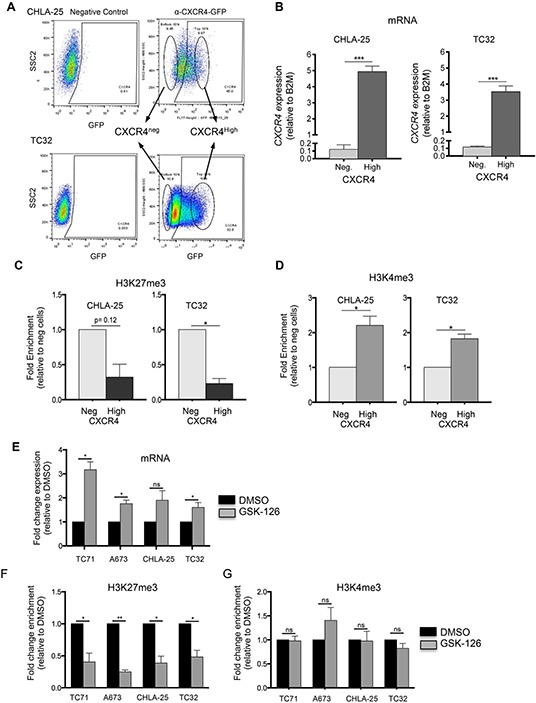
Upregulation of *CXCR4* is associated with loss of H3K27me3 **A.** CHLA-25 and TC32 cells were FACS-sorted into CXCR4^Neg^ (bottom 10%) and CXCR4^High^ (top 10%) populations. **B.** qRT-PCR confirmed that expression of *CXCR4* mRNA correlated with CXCR4 surface protein expression in sorted cells. **C.** ChIP-qPCR experiments revealed that the H3K27me3 modification is relatively reduced in CXCR4^High^ populations compared to CXCR4^Neg^ cells. **D.** In contrast to H3K27me3, the H3K4me3 mark was shown to be increased in CXCR4^High^ populations. **E.** Exposure of Ewing sarcoma cells to GSK-126 resulted in an increase in *CXCR4* expression. **F.** GSK-126 treated cells showed a loss of H3K27me3 at the *CXCR4* promoter **G.** GSK-126 had no impact on H3K4me3 enrichment. Data represented as mean ± SEM of three independent experiments. *. P <0.05 as compared to controls.

Consistent with a poised, bivalent chromatin state, most Ewing sarcoma cells do not express high levels of *CXCR4* [9]. However, we previously noted that two Ewing sarcoma cell lines, CHLA-25 and TC-32, exist in an equilibrium state wherein 30-40% of cells express high levels of CXCR4 transcript and protein [9]. We took advantage of the inherent heterogeneity of these two cell lines to directly address the contribution of the H3K27me3 modification to *CXCR4* gene repression and H3K4me3 modification to gene activation. CHLA-25 and TC-32 cells were FACS-sorted to isolate pure populations of CXCR4 negative and CXCR4 positive populations (Figure 5A). As expected, *CXCR4* mRNA expression in these populations correlated with surface protein expression (Figure 5B). Moreover, analysis of histone modifications demonstrated preferential enrichment of H3K27me3 in the CXCR4 negative populations (Figure 5C) and H3K4me3 in the CXCR4 high populations (Figure 5D), supporting a role for these modifications in gene regulation. The H3K27me3 modification is mediated by the polycomb repressive complex protein EZH2, which functions as a histone methyltransferase and as a pro-tumorigenic oncogene in Ewing sarcoma [16]. To determine if the presence of H3K27me3 directly mediates silencing of *CXCR4* gene expression we exposed cells to GSK-126, a highly selective pharmacologic inhibitor of EZH2 methyltransferase activity [17]. Expression of *CXCR4* increased in all Ewing sarcoma cell lines following exposure to GSK-126 (Figure 5E) and this was accompanied by loss of the H3K27me3 modification (Figure 5F) but no change in H3K4me3 enrichment at the *CXCR4* promoter (Figure 5G). Thus, repression of *CXCR4* expression in Ewing sarcoma is, at least in part, dependent on EZH2 and on the presence of the H3K27me3 modification at the gene promoter.

**Figure 6:**
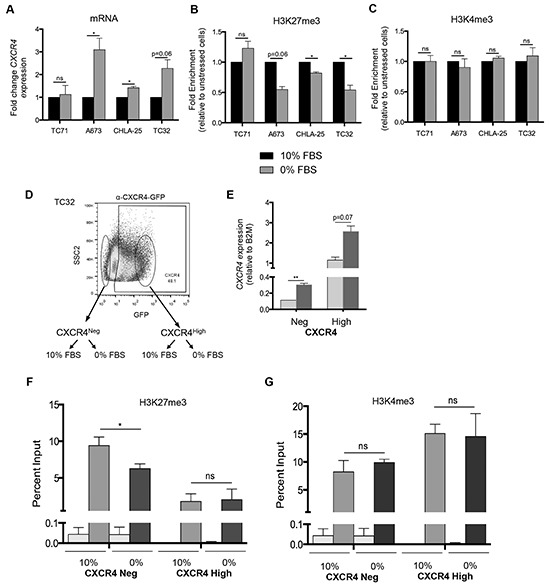
Ewing sarcoma cells lose the repressive H3K27me3 mark at the CXCR4 promoter in response to stress **A.** qRT-PCR shows that serum starvation resulted in upregulated expression of *CXCR4* expression in three of four Ewing sarcoma cell lines. **B.** CHIP-qPCR confirmed diminished H3K27me3 enrichment at the *CXCR4* promoter in cells with upregulated transcript expression. **C.** No change in H3K4me3 enrichment was induced by serum deprivation. **D.** TC32 cells were FACS-sorted to isolate CXCR4 negative cells which were then placed into 10% FBS or serum deprived conditions for 24 hours. **E.** *CXCR4* mRNA expression was induced by serum deprivation in CXCR4 negative (p<0.005) and also in CXCR4 positive cells (p=0.07) **F.** Loss of H3K27me3 was evident in CXCR4 negative but not CXCR4 positive cells following serum deprivation. **G.** Serum withdrawal had no impact on H3K4me3 enrichment at the *CXCR4* promoter in either CXCR4 negative or CXCR4 positive cells. Data are represented as mean ± SEM (n=3 for *CXCR4* expression, n=2 or 3 for ChIP experiments). *. P <0.05 ** P <0.005 as compared to controls.

Finally, we evaluated whether changes in these histone modifications were evident in Ewing sarcoma cells that had been exposed to microenvironmental stress. Withdrawal of serum for 24 hours resulted in upregulated expression of *CXCR4* in three of four cell lines (Figure 6A) and in all three cases this was reproducibly accompanied by loss of H3K27me3 at the gene promoter (Figure 6B). In contrast, no change in H3K4me3 was observed (Figure 6C). To further define the nature of cells that experienced loss of bivalency in the context of growth factor withdrawal, we FACS-sorted TC-32 cells on the basis of CXCR4 expression and exposed the sorted cells to either 10% serum or serum free media for 24 hours (Figure 6D). Interestingly, *CXCR4* transcript expression was relatively increased in both populations of cells that were subjected to serum withdrawal although the increase in CXCR4 positive cells was not statistically significant (Figure 6E). Concomitant with transcript upregulation, CXCR4 negative cells showed a loss of H3K27me3 enrichment at the *CXCR4* promoter (Figure 6F) while enrichment of H3K4me3 was unchanged (Figure 6G). No change in either mark was detected in CXCR4 positive cells following serum withdrawal indicating that the increase in transcript was not mediated by a loss of H3K27me3 (Figure 6F & 6G).

**Figure 7:**
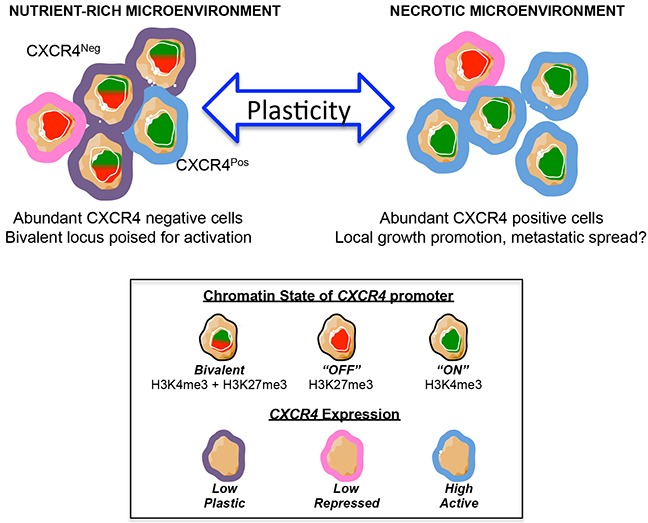
Model of CXCR4 plasticity Schematic model depicts the potential implications of our results. In this model, stress – such as a necrotic tumor microenvironment – induces CXCR4 negative tumor cells to convert into CXCR4 positive cells, which promote tumor progression *via* activation of the CXCL12/CXCR4 signaling axis. This conversion is, at least in part, mediated by epigenetic switching of the *CXCR4* promoter from an inactive bivalent state to a univalent active state.

Thus, these data together reveal that CXCR4 negative Ewing sarcoma cells exist in a bivalent state wherein the *CXCR4* promoter is repressed but poised for rapid activation. Exposure of bivalent tumor cells to microenvironmental stress, such as growth factor deprivation, results in loss of bivalency and gene activation, thereby contributing to stress-dependent induction of *CXCR4* and phenotypic transition of cells from CXCR4 negative to CXCR4 positive states (Figure 7).

## DISCUSSION

Ewing sarcomas are aggressive bone and soft tissue tumors that are characterized by the presence of pathognomonic chromosomal translocations that most commonly result in creation of an *EWS-FLI1,* or related *EWS-ERG,* oncogenic fusion gene [18]. Deep sequencing studies of primary Ewing sarcoma tumors at the time of diagnosis have shown that recurrent mutations outside of the tumor-defining fusion are infrequent, demonstrating that clonal, genetic heterogeneity is uncommon prior to therapy [19–21]. In contrast, disruptions to the normal epigenome and epigenetic regulatory complexes are prevalent in Ewing sarcoma and have been shown to be central to tumor pathogenesis [16, 22–27]. Moreover, Ewing sarcomas are presumed to arise from mesenchymal and/or neural crest stem or progenitor cells which by nature are epigenetically plastic [2, 28]. Thus, it is likely that epigenetic plasticity plays a key role in mediating Ewing sarcoma tumor cell heterogeneity.

Phenotypic heterogeneity among Ewing sarcoma tumor cells has, to date, been relatively understudied. The characteristic small, round, blue cell histology of these tumors is most commonly associated with a very homogeneous cellular morphology, uniform membranous expression of CD99, and an absence of differentiation markers [29]. In addition, clinical standard of care provides only very small needle biopsies prior to the initiation of neoadjuvant chemotherapy, making robust studies of heterogeneity in large tumor resections impossible [30, 31]. Thus most studies of cellular heterogeneity in Ewing sarcoma have, to date, focused on defining inter-tumoral differences in oncogenic fusion type, secondary genetic alterations, gene expression profiles, and clinical behavior [18–21, 30, 32, 33]. Nevertheless, a number of studies of both primary tumor specimens and Ewing sarcoma cell lines have shown that substantial phenotypic heterogeneity exists among individual tumor cells and direct and indirect evidence from these studies supports the conclusion that this heterogeneity contributes to differences in tumorigenicity, progression, metastatic potential, and treatment response [34–37].

In the current study, we confirmed that Ewing sarcoma cells convert between CXCR4 negative and CXCR4 positive states *in vivo.* In addition, we observed that CXCR4 negative cells had a propensity to generate CXCR4 positive cells adjacent to areas of tumor necrosis. Thus, these data support our prior *in vitro* data that CXCR4 negative cells can be converted into CXCR4 positive states cells in response to microenvironmental stress [9]. In addition, we detected a direct correlation between tumor size and *CXCR4* expression at the time of necropsy. This finding could indicate that rapidly growing tumors upregulate *CXCR4* as they outstrip their blood supply. Alternatively, the positive correlation between *CXCR4* expression and tumor size might be due to the direct impact of CXCR4 signaling on tumor growth. Indeed, a prior study showed that CXCL12 promotes the proliferation of CXCR4 positive Ewing sarcoma cells *in vitro* and the same study also identified a positive correlation between tumor volume and the presence of CXCR4 positive tumor cells in primary human tumors [11]. Thus, CXCR4 positive tumor cells are likely to play a fundamental role in local progression of Ewing sarcoma. Whether or not these cells also contribute to tumor metastasis remains to be determined. Our finding that rates of engraftment are equivalent, in a tail vein model, between CXCR4 negative and CXCR4 positive cells will be informative for future studies of CXCR4 function in models that can evaluate other downstream steps in the metastatic cascade.

From a mechanistic perspective, we found that phenotypic heterogeneity of CXCR4 in Ewing sarcoma tumor cells is, in part, epigenetically mediated. The *CXCR4* promoter of CXCR4 negative cells exists in a bivalent state under ambient conditions, characteristic of a transcriptionally silent locus that is poised for activation [13]. When CXCR4 negative Ewing sarcoma cells are deprived of growth factors they upregulate *CXCR4* transcript expression and we observed that this is associated with loss of the repressive H3K27me3 modification and retention of the activating H3K4m3 modification. In addition, pharmacologic inhibition of EZH2 leads to increased expression of *CXCR4* and loss of H3K27me3, providing further evidence that this epigenetic modification contributes to transcriptional repression of the locus in non-stressed conditions. High levels of EZH2 in Ewing sarcoma cells contribute to maintenance of the tumorigenic state and this is mediated, at least in part, by EZH2-mediated repression of cell differentiation [16, 27]. Thus, EZH2 inhibition has been discussed as a potential therapeutic target. However, given our current findings, it will be important to consider that use of EZH2 inhibitors as anti-cancer agents in Ewing sarcoma could have the on-target, but undesirable, effect of activating CXCR4. Further investigation into the potential negative impact of CXCR4 activation on tumor progression is warranted in preclinical studies of EZH2-inhibition.

Studies of breast cancer recently demonstrated the importance of promoter bivalency to phenotypic plasticity and tumor cell heterogeneity [14]. In particular, Chaffer *et al.* showed that, in sub-populations of breast cancer cells, the promoter of *ZEB1*, a master regulator of epithelial-to-mesenchymal transition, is maintained in an inactive, bivalent state and that loss of bivalency in these cells is induced in response to TGFβ [14]. Importantly, the bivalent state of the *ZEB1* promoter defined cell populations that were able to convert from non-cancer stem cell to cancer stem cell states as a result of their ability to resolve the *ZEB1* locus into an activate chromatin configuration [14]. In contrast, non-cancer stem cells whose *ZEB1* promoter existed in a univalent, H3K27me3 marked state were unable to activate either *ZEB1* transcription or a cancer stem cell phenotype in response to TGFβ [14]. Thus, promoter bivalency was shown to be a key mediator of cell plasticity and the breast cancer stem cell phenotype. These data, combined with our own, now raise the intriguing possibility that bivalency of the *CXCR4* locus may be an important factor that contributes to the conversion of CXCR4 negative breast cancer stem cells into CXCR4 positive metastasis-inducing breast cancer stem cell populations [38]. The contribution of epigenetic plasticity to CXCR4 heterogeneity is now worthy of investigation in the context of other tumors that display stem cell characteristics and in which CXCR4 positive cells contribute to tumor progression.

Finally, it is clear from the current work, and from our prior studies, that not all Ewing sarcoma cells uniformly upregulate CXCR4 in response to stress. Some tumor cell lines, such as TC-32 and CHLA-25, are highly plastic and responsive to stress, whereas others, such as TC71, are generally unresponsive. TC71 cells did not activate *CXCR4* transcription under conditions of serum deprivation. Nevertheless, exposure of TC71 cells to a potent pharmacologic inhibitor of EZH2 did result in gene activation concomitant with loss of H3K27me3 and ChIP studies confirmed promoter bivalency. Thus, the bivalent state of the *CXCR4* promoter in TC71 cells contributes to gene silencing and is retained under conditions of serum deprivation. Studies of DNA methylation failed to identify differences in promoter DNA methylation among the different cell lines (not shown), supporting the conclusion that other, as yet unknown mechanisms, contribute to *CXCR4* repression in TC71 cells. Given what is known about the complexity of CXCR4 regulation, from transcriptional to post-transcriptional mechanisms, translational to post-translational modifications, and the active process of sub-cellular trafficking [39, 40], it is highly likely that multiple mechanisms contribute to dynamic regulation of CXCR4 in Ewing sarcoma. Our own observation that CXCR4 positive TC-32 cells further upregulate *CXCR4* expression upon serum withdrawal demonstrates that loss of promoter bivalency is not the sole mechanism driving stress-dependent activation. In addition, recent identification of *CXCR4* splice variants in Ewing sarcoma tumors and cell lines raises the possibility that alternate promoter usage and altered post-transcriptional regulation might also contribute to dynamic regulation and expression of CXCR4 [41]. Further studies are needed to fully elucidate which of these many mechanisms are activated in response to different microenvironmental cues and how they contribute to tumor progression.

Tumor heterogeneity continues to be a major impediment to cancer cures. In the context of Ewing sarcoma, epigenetic plasticity is likely to play a major role in mediating this heterogeneity and promoting disease progression. Continued elucidation of the mechanisms by which Ewing sarcoma cells alter their phenotypes to adopt more aggressive states is warranted so that novel approaches to therapy can be developed that specifically target and inhibit these heterogeneity-inducing processes.

## MATERIALS AND METHODS

### Cell lines

Ewing sarcoma cell lines were cultured in RPMI-1640 media (Gibco, Grand Island, NY, USA) supplemented with 10% FBS (Atlas Biologicals, Inc., Fort Collins, CO, USA) and 6mM L-glutamine (Life Technologies, Grand Island, NY, USA) at 37°C and 5% CO_2_. For CHLA-25 cells, prior to cell seeding, plates were briefly coated (~5 minutes) with 0.2% Gelatin (Gelatin from bovine skin, Type B). For serum starvation conditions, cells were cultured as above without the presence of FBS for 24 hours. For hypoxia studies, cells were incubated inan xVivo system (Biospherix, Lacona, NY, USA) at 1% O_2,_ 37°C and 5% CO_2_ for 48 hours. For GSK-126 studies, cells were treated with either vehicle control (DMSO; D128-500, Fisher Scientific, Waltham, MA) or 10μM GSK-126 (A-1275, Active Biochem, Maplewood, NJ) daily for 72 hours prior to functional studies.

### Immunohistochemistry

Immunohistochemistry for CXCR4 and CD99 was performed on formalin fixed paraffin embedded tumor sections using the Dako Autostainer Link (Dako, Carpinteria, CA). Following rehydration, sections were treated with heat induced epitope retrieval (HIER). For CXCR4 HIER was performed with FLEX TRS Low pH Retrieval buffer (pH 6.10) (Dako) for 20 minutes. For CD99 HIER was performed with FLEX TRS High pH Retrieval buffer (pH 9.01) (Dako) for 20 minutes. After peroxidase blocking, CXCR4 antibody (ab124824; abcam, Cambridge, MA) was applied at a dilution of 1:4000 at room temperature for 60 minutes, and CD99 antibody (M3601; Dako, Carpinteria, CA) was applied at a dilution of 1:100 at room temperature for 60 minutes. The FLEX HRP EnVision System (Dako) was used for detection. DAB chromagen was then applied for 10 minutes. Slides were counterstained with Harris Hematoxylin for 5 seconds and then dehydrated and coverslipped.

### Quantitative real-time PCR

RNA was isolated using the Quick-RNA™ MiniPrep kit (Zymo Research, Irvine, CA) and cDNA was generated using iScript (Bio-Rad, Hercules, CA). Quantitative real-time PCR was performed using validated *CXCR4* and beta-2-microglobulin (*B2M*) Taqman assays (Life Technologies, Grand Island, NY). Analysis was performed in triplicate using the Lightcycler® 480 System. Using the ΔΔCt method, gene expression was normalized to the reference gene.

### Chromatin immunoprecipitation (ChIP)

Chromatin immunoprecipitation was performed according to the methods of Gilfillan et al. 2012 [42]. In brief, Ewing sarcoma cells (3.6×10^5^ per IP) were digested with Micrococcal nuclease (MNase) (70196Y, Affymetrix, Santa Clara, CA) for 5 minutes at 37°C, sonicated for 20 seconds (Qsonica cup horn sonicator (Qsonica Sonicators, Newtown, CT, USA)), blocked for 1 hour with Dynabeads A+G (10001D and 10003D; Life Technologies, Carlsbad, CA), incubated with 1 μg of desired antibody overnight, incubated with Dynabeads A+G for 3 hours, washed (5 minute wash; 5 x RIPA buffer, 1 x LiCl buffer, 1 x TE buffer), digested proteins with Proteinase K for 1 hour at 55°C and purified immunoprecipitated DNA according to manufacturer's instructions (Zymo Genomic DNA Clean & Concentrator, D4011). For ChIP-re-ChIP studies, after the 3 hour incubation with Dynabeads A+G, the beads were incubated with dithiothreitol (DTT) (10mM final concentration, 15508-013, Life Technologies, Carlsbad, CA) for 30 minutes at 37°C. Chromatin was then incubated with the desired second antibody overnight and the protocol continued as above. Primer pairs for the *CXCR4* promoter region are listed in Supplementary Table S1. ChIP antibodies were used as per manufacturer's instructions; H3K4me3 Rabbit anti-Human Polyclonal Antibody (49-1005; Life Technologies, Carlsbad, CA), Anti-trimethyl-Histone H3 (Lys27) Antibody (07-449; Millipore, Billerica, MA), normal mouse IgG (sc-2025; Santa Cruz Biotechnology, Dallas, TX), Rabbit IgG (ab37415; Abcam, Cambridge, MA).

### Cell sorting

Cell sorting was performed as previously described [9]. In brief, cells were blocked for 15 minutes at 4°C with agitation (0.5% FBS), incubated with human CXCR4 Alexa Fluor 488 or Alexa Fluor 700 monoclonal antibody (5 uL/1.0×10^6^ cells) (R&D Systems, Minneapolis, MN) for 30 minutes at 4°C with agitation, passed through a 0.40 μm sterile nylon mesh strainer and sorted into CXCR4^neg^ and CXCR4^high^ on a Beckman Coulter MoFlo Astrios.

### In vivo xenografts

GFP/Luciferase tagged TC-32 cells were first sorted by FACS into CXCR4^neg^ and CXCR4^high^ populations and 1 million sorted cells were injected into NOD SCID mice. Bioluminescence imaging of mice was performed on the Perkin Elmer *In Vivo* IVIS Spectrum Optical Imaging System to assess time to engraftment post-injection (Center for Molecular Imaging Core, University of Michigan). All animal studies were performed in accordance with protocols approved by the University of Michigan Animal Care and Use Committee.

### Statistical analysis

Data are reported as mean ± SEM from at least three independent experiments and p-values calculated using ratio paired t-test unless otherwise indicated. P-values of <0.05 were considered significant.

## SUPPLEMENTARY FIGURE AND TABLE


