# Supplementary material for: A bivalent promoter contributes to stress-induced plasticity of CXCR4 in Ewing sarcoma
Source: Oncotarget. 2016 Aug 12;7(38):61775–88. doi: 10.18632/oncotarget.11240 (PMC5308690; doi:10.18632/oncotarget.11240)
Supplement: Supplementary file 1 [file oncotarget-07-61775-s001.pdf]

A bivalent promoter contributes to stress-induced plasticity of CXCR4 in Ewing sarcoma

SUPPLEMENTARY FIGURE AND TABLE

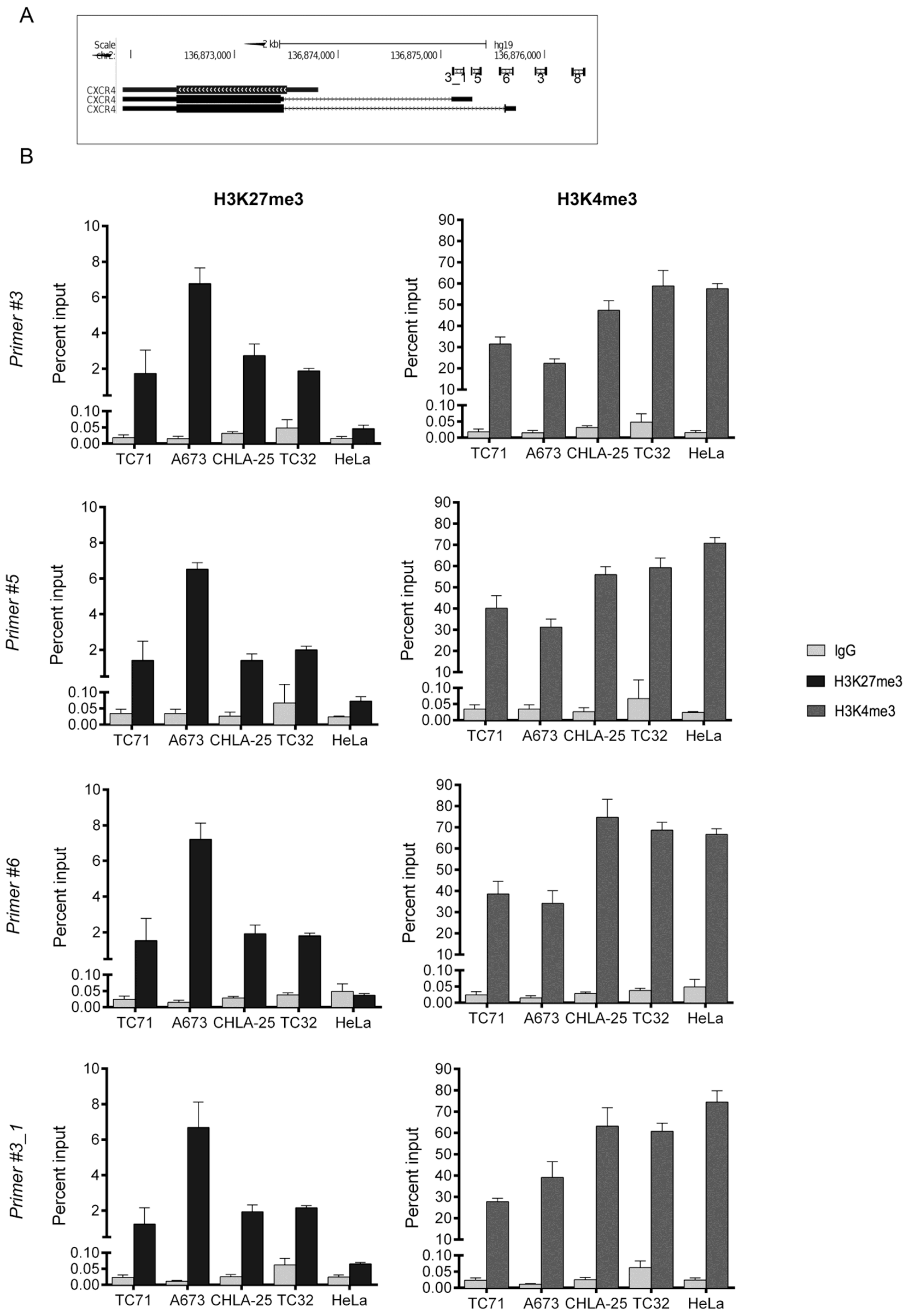

Supplementary Figure S1: Additional primer pairs for directed ChIP-PCR of *CXCR4* promoter. A. Map of primer pairs designed for the *CXCR4* promoter region. Primer pair #8 was used for studies shown in main figures **B.** ChIP-qPCR using additional primers confirmed enrichment of H3K4me3 and H3K27me3 at the *CXCR4* promoter in Ewing sarcoma cells and only H3K4me3 in HeLa cells. Data represented as mean ± SEM of three independent experiments.

Supplementary Table S1: PCR primer sequences

**CXCR4 Promoter**			
**Primer pair**	**Forward**	**Reverse**	**Size (bp)**
#3	5′- GGG TGG TCG GTA GTG AGT CC -3′	5′- CAG AGA GAC GCG TTC CTA GC -3′	120
#5	5′- ACG CCT TCT CTG CAG TTG TG -3′	5′- TTC CAG TGG CTG CAT GTG TC -3′	105
#6	5′- GGG CTG CGC TCT AAG TTC AA -3′	5′- TAG CAA AGT GAC GCC GAG G -3′	144
#3_1	5′- GGG ATG TCT TGG AGC GAG TT -3′	5′- AAC AGT CAC CAG GCG CTT AA -3′	122
#8	5′- TCA CTA GGG TCA GGT GCA GA -3′	5′- TCG CGA ATT GGT TAC CGC TA -3′	131
